# Association between dietary phytochemical index and 3-year changes in weight, waist circumference and body adiposity index in adults: Tehran Lipid and Glucose study

**DOI:** 10.1186/1743-7075-9-108

**Published:** 2012-12-03

**Authors:** Parvin Mirmiran, Zahra Bahadoran, Mahdieh Golzarand, Niloofar Shiva, Fereidoun Azizi

**Affiliations:** 1 Department of Clinical Nutrition and Dietetics, Faculty of Nutrition Sciences and Food Technology, National Nutrition and Food Technology Research Institute, Shahid Beheshti University of Medical Sciences, Tehran, Iran; 2 Obesity Research Center, Research Institute for Endocrine Sciences, Shahid Beheshti University of Medical Sciences, Tehran, Iran; 3 Endocrine Research Center, Research Institute for Endocrine Sciences, Shahid Beheshti University of Medical Sciences, Tehran, Iran

**Keywords:** Dietary phytochemical index, Waist circumference, Body adiposity index, Weight gain, Tehran lipid and Glucose study

## Abstract

**Background:**

High intakes of phytochemical-rich foods have favorable effects on the prevention of chronic diseases. In this study we assessed the dietary phytochemical index (PI) in relation to 3-year change in weight, waist circumference (WC), body adiposity index (BAI) among Tehranian adults.

**Methods:**

This longitudinal study was conducted in the framework of Tehran Lipid and Glucose Study, between 2006–2008 and 2009–2011, on 1938 adults, aged 19–70 y. The usual intake of participants was measured at baseline using a validated semi-quantitative food frequency questionnaire and dietary PI was calculated. Anthropometric measures were assessed both at baseline and 3 years later. Multiple regression models were used to estimate mean difference changes in anthropometrics associated with various dietary PI.

**Results:**

The mean age of participants was 40.4 ± 13.0 y, at baseline, respectively. Mean weight gain was 1.49 ± 5.06 kg (1.65 ± 5.3 kg in men and 1.34 ± 4.9 kg in women) during 3-year period. After adjustment for potential confounding variables including age at baseline, sex, BMI, educational levels, smoking, physical activity, total energy intake, dietary intake of carbohydrate, fat and protein, dietary intakes of whole grains in the highest quartile category of PI were inversely associated with 3-year changes in weight and WC (*P for trend <0.05*). Dietary intake of fruits in the highest quartile was also associated with lower weight gain during the study period (*P for trend <0.05*). There was significant inverse association between the highest quartile category of dietary PI with the 3-year changes in weight and BAI (*P for trend <0.05*).

**Conclusion:**

Higher dietary PI could have favorable effects on prevention of weight gain and reduction of body adiposity in adults.

## Background

Obesity and abdominal obesity are major public health problems worldwide, and are associated with increased risk of non-communicable diseases, morbidity and mortality [1-3]. The global prevalence of obesity has doubled between 1980 and 2008. In 2008, 1.5 billion adults, aged 20 year and older, were overweight and 500 million were obese worldwide, figures estimated to reach 2.3 billion (overweight) and over 700 million (obese) in 2015 [4,5]. Parallel to the ascending trend of overweight and obesity worldwide, the prevalence of overweight and obesity is increasing in Iran; one study reported that the estimated total prevalence of obesity and abdominal obesity in 1999–2001 were 15.8 and 36.5% in men and 31.3 and 76.7% in Iranian women, respectively [6]. An increasing trend in the prevalence of obesity and abdominal fat gain was also observed among adults during the last years [7].

Obesity is a multi-factorial disease and dietary intake is the major modifiable factor which may be responsible for the increasing trend in overweight and obesity. Several studies have documented that plant foods and plant-based diets like vegetarian diets are associated with the prevention and treatment of chronic diseases [8-10]. Epidemiological and cross-sectional studies have demonstrated that vegetarian diets have an inverse association with weight and body mass index (BMI) [11-14] and a recent meta-analysis conducted on 60 studies has shown that vegetarians had significantly lower weight and BMI compared with non-vegetarians [15]. Others studies have been shown that high intakes of fruits, vegetables, whole grains and legumes have inverse associations with obesity and weight gain and recommend high consumption of these food groups for prevention obesity and maintenance of weight [16-18].

There is growing evidence that the beneficial effects of whole plant foods, in addition to lower energy density and glycemic index, may be contributed by the many phytochemicals found in these food groups. Phytochemicals are natural non-nutritive bioactive compounds including phenolic compounds (flavonoids, phenolic acids, hydroxycinammic acids, lignans, tyrosol esters, stilbenoids), isoperenoids, organosulfor compounds (allyl sulfors, isothiocyanates) found in fruits, vegetables, whole grains, nuts, legumes and other plant foods [19-21]. These phytochemicals in addition to direct antioxidant activity, their anti-inflammatory potential and the modulation of carbohydrate and lipid metabolism, have anti-obesity properties [21].

In this population based longitudinal study, we assessed the baseline dietary phytochemical index (PI) developed by McCarty as an index of total dietary phytochemical content, in relation to 3-year changes in weight, waist circumference and body adiposity index (BAI) among Tehranian adults.

## Methods

### Study population

This study was conducted within the framework of the Tehran Lipid and Glucose Study (TLGS). Briefly, TLGS is a community-based prospective study conducted to investigate and prevent non-communicable diseases, in a representative sample of residents, aged ≥ 3y, from district 13 of Tehran, the capital city of Iran. The first phase of the TLGS began in March 1999 and data collection, at three-year intervals, is ongoing [22,23].

For the current study, 2799 men and women aged 19–70 y, were recruited. Participants were excluded if they under- or over reported dietary intakes (less than 800 kcal/d or more than 4200 kcal/d, respectively), or they were on specific diets. The final sample at baseline (2006–2008), included 2567 adults (1129 men and 1438 women). The mean duration of the follow-up was approximately 3 years. Of the 2567 initial participants who attended the baseline examination, 629 participants who had no follow-up information on anthropometric measurements were excluded and final analysis was performed in 1938 (75.5%) participants.

Informed written consents were obtained from all participants and the study protocol was approved by the research council of the Research Institute for Endocrine Sciences, Shahid Beheshti University of Medical Sciences.

### Dietary assessment and phytochemical index calculation

Dietary data were collected using a validated semi-quantitative FFQ with 168 food items. Trained dietitians with at least 5 years of experience in TLGS survey asked participants to designate their intake frequency for each food item consumed during the past year on a daily, weekly, or monthly basis. Portion sizes of consumed foods reported in household measures were then converted to grams [23]. The validity and reliability of the FFQ were assessed in a random sample (based on sex and age groups), by comparing the data from two FFQs completed 1 y apart and comparing the data from the FFQs and multiple 24-hour dietary recalls, respectively. The validity and reliability of the FFQ for dietary intakes were acceptable; the correlation coefficients between the FFQ and multiple 24 recalls were 0.59 and 0.38 and those between the two FFQs were 0.43 and 0.42 in male and female participants, respectively.

Because the Iranian Food Composition Table (FCT) is incomplete, and has limited data on nutrient content of raw foods and beverages, to analyze foods and beverages for their energy and nutrient content we used the U.S. Department of Agriculture (USDA) FCT (23).

The dietary phytochemical index was calculated based on method developed by McCarty; [PI = (daily energy derived from phytochemical-rich foods kcal/total daily energy intake kcal) × 100] [24]. Foods included in the phytochemical-rich category were fruits and vegetables, legumes, whole grains, nuts, soy products, olives and olive oil. Potatoes were not considered as vegetables because they are often consumed as a starch component rather than as vegetables. Natural fruit and vegetable juices as well as tomato sauces were included in the fruit and vegetable groups because these are also considered as rich sources of phytochemicals.

### Lifestyle and anthropometric measurement

Trained interviewers collected information using a pretested questionnaire. Information on age (years), current smoking (yes/no), educational level (illiterate, primary, academic and advanced academic education) and physical activity (MET-h/wk) were assessed at baseline examination (2006–2008). Smoking status was obtained using face-to-face interviews and participants who smoked daily or occasionally were considered current smokers, and non-smoker included those who had never smoked or those who cessation smoking. Physical activity level was assessed using the Persian translated Modifiable Activity Questionnaire (MAQ) [25]. The frequency and time spent on light, moderate, hard and very hard intensity activities according to the list of common activities of daily life over the past year were obtained. Physical activity levels were expressed as metabolic equivalent hours per week (METs h/wk). High reliability and relatively moderate validity were reported for the Persian translated MAQ in Tehranian adults [26]. Anthropometric measurements were assessed at baseline by trained staff. Weight was measured to the nearest 100 g using digital scales, while the participants were minimally clothed, without shoes. Height was measured to the nearest 0.5 cm, in a standing position without shoes, using a tape meter. Waist circumference (WC) and hip were measured to the nearest 0.1 cm, at anatomical landmarks, at the widest portion, over light clothing, using a soft, cloth tape meter, without any pressure to the body. Body mass index was calculated as weight (kg) divided by square of the height (m^2^). Body adiposity index was calculated as [hip(cm)/height(m) ^1.5^-18 [27]. All measurements at follow-up (2009–2011) were again obtained by the same protocol used during baseline examinations.

### Statistical methods

All statistical analysis were conducted using SPSS (Version 16.0; Chicago, IL), and *P* values < 0.05 was considered significant. Participant characteristics were compared across quartile categories of PI, adjusted for sex and age, using the general linear model or the Chi-square test. Mean dietary intakes of participants were compared across quartile categories of PI using the general linear model with adjustment for sex and age. P values for linear trend between the PI score as continuous variables and participant characteristics and dietary intakes as were assessed using linear regression for continuous characteristics and logistic regression for dichotomous characteristics.

Changes of anthropometric measures, during 3-year follow-up, were calculated as [(follow-up measure - baseline measure)/baseline measure] × 100. Multiple regression models were used to evaluate the association between dietary PI and% of changes in weight, WC and BAI. Dietary PI was categorized into quartiles (<20.9, 20.9-28.3, 28.4-37.1 and >37.1). Participants with dietary PI < 20.9 were considered as the reference group. To determine the association between each phytochemical-rich food groups and 3-year changes in anthropometric measures, we also categorized energy adjusted intakes of whole grains, vegetables, fruits, legumes, nuts, soy, olives and olive oil, into quartiles. Mean changes percent of all anthropometric measures associated with each category of dietary PI or phytochemical-rich food, compared with the reference group and their 95% confidence intervals were estimated using the multiple regression models with adjustment for potential confounding variables. The variables adjusted in the models were sex, age at baseline (y, continuous), BMI (kg/m^2^, continuous), education (4 categories), smoking (yes or no), physical activity (MET-h/wk, continuous), total energy intake (kcal/d), dietary carbohydrate (% of energy), fat (% of energy) and protein (% of energy). A linear trend test was performed considering each ordinal score variable as a continuous variable in the model.

## Results

The mean age of participants was 40.4 ± 13.0 y, and mean BMI was 27.03 ± 4.9 kg/m^2^ at baseline; forty-seven percent of participants were men. The mean weight gain was 1.49 ± 5.06 kg (1.65 ± 5.3 kg in men and 1.34 ± 4.9 kg in women) during the 3-year period. The means for WC of participants at baseline and 3-year change in WC were 89.6 ± 13.2 cm (94.8 ± 10.8 in men, and 85.6 ± 13.6 in women), and 4.3 ± 6.9 cm (1.8 ± 4.8 in men, and 6.3 ± 7.7 in women), respectively. The means for BAI at baseline and BAI change during 3 years were 30.6 ± 6.3% (26.0 ± 3.4 in men, and 34.2 ± 5.8 in women), and −0.02 ± 2.28% (0.42 ± 1.71 in men, and −0.37 ± 2.5 in women), respectively. The mean PI was 29.8 ± 12.3 (28.5 ± 12.1 in men, and 30.9 ± 12.3 in women), and dietary PI ranged from 20.9 to 37.1 and was associated with several participant characteristics (Table 1). Participants in the highest PI quartile category were more likely to be women than men (65.2 *vs.* 34.8%, *P for trend <0.05*), were older (36 *vs.* 46 years, *P for trend <0.001*), and were less likely to be current smokers (8.2 *vs.* 14.5%, *P for trend <0.01*). They also had lower weight (71.6 *vs.* 74.3 kg, *P for trend <0.05*), and waist circumference (88.3 *vs.* 91.1 cm, *P for trend <0.001*) at baseline, and lower weight (72.9 *vs.* 75.9 kg, *P for trend <0.05*) at the follow up. The three-year weight gain in participants with highest dietary PI was significantly lower than those in the lower quartile of PI (0.9 *vs.* 1.9 kg, *P for trend <0.05*).

**Table 1:** **Characteristics of participants by categories of dietary phytochemical index: Tehran Lipid and Glucose Study**^**1**^

	**(*n *= 1938)**				
	***Q1***	***Q2***	***Q3***	***Q4***	***P***^***1***^
Dietary phytochemical index					
* Range*	<20.9	20.9–28.3	28.4–37.1	>37.1	
* Mean*	17.4 ± 5.6	25.4 ± 6.4	32.9 ± 8.3	42.7 ± 10.7	
Age at baseline *(yr)*	36.4 ± 0.5	38.2 ± 0.5	40.5 ± 0.5	46.0 ± 0.5	*<0.001*
Men *(%)*	55.9	44.4	40.2	34.8	*<0.05*
Weight *(kg)*					
* Baseline*	74.3 ± 0.6	73.3 ± 0.6	71.9 ± 0.6	71.6 ± 0.6	*0.015*
* After 3 year*	75.9 ± 0.6	74.5 ± 0.6	73.6 ± 0.6	72.9 ± 0.6	*0.017*
* 3-year change*	1.9 ± 0.2	1.4 ± 0.2	1.7 ± 0.2	0.9 ± 0.2	*0.017*
Waist circumference *(cm)*					
* Baseline*	91.1 ± 0.5	90.0 ± 0.5	89.1 ± 0.5	88.3 ± 0.5	*0.003*
* After 3 year*	94.7 ± 0.5	94.0 ± 0.5	93.7 ± 0.5	93.2 ± 0.5	*0.24*
* 3-year change*	4.1 ± 0.3	4.2 ± 0.3	4.6 ± 0.3	4.3 ± 0.3	*0.74*
Body adiposity index *(%)*					
* Baseline*	30.4 ± 0.2	30.4 ± 0.2	30.6 ± 0.2	30.8 ± 0.2	*0.52*
* After 3 year*	30.5 ± 0.1	30.5 ± 0.1	30.7 ± 0.1	30.6 ± 0.1	*0.23*
* 3-year change*	1.09 ± 0.32	0.28 ± 0.32	0.69 ± 0.32	−0.38 ± 0.32	*0.01*
Physical activity *(Met-h/week)*					
* Job activity*	30.7 ± 2.4	26.3 ± 2.3	24.6 ± 2.3	23.2 ± 2.4	*0.17*
* Leisure time activity*	9.8 ± 0.8	9.5 ± 0.7	10.0 ± 0.7	11.2 ± 0.7	*0.43*
* Total*	40.5 ± 2.6	35.7 ± 2.4	34.6 ± 2.4	34.4 ± 2.5	*0.31*
Current smoker (%)	14.5	12.6	9.6	8.2	*0.01*
Education status *(%)*					
* Illiterate*	2.0	1.6	1.8	3.7	*0.11*
* Primary education*	7.9	6.4	0.0	5.6	*0.81*
* Academic education*	84.2	83.0	91.4	83.3	*0.81*
* Advanced academic education*	7.9	10.6	8.6	11.1	*0.81*

^1^ Data are age-adjusted mean ± SEM.

^2^*P* values compared the characteristics of participants across quartiles of dietary phytochemical index using Chi square test or linear regression models with adjustment of sex and age.

The mean dietary intake of participants across dietary PI quartile categories is presented in Table 2. Dietary energy and fat intakes decreased significantly across quartiles of PI (*P for trend <0.001*), while dietary intakes of carbohydrate, protein, fiber, vitamin E and vitamin C increased significantly (*P for trend <0.001*). Dietary intakes of whole grains, fruits, vegetables and legumes in the highest quartile category of PI, were respectively 25, 3.5, 1.7 and 1.5 times higher compared with lower quartile categories.

**Table 2:** **Mean dietary intake of participants by categories of dietary phytochemical index at baseline (2006–2008)**^**1**^

	**(*n*= 1938)**				
	***Q1***	***Q2***	***Q3***	***Q4***	***P***^***1***^
Dietary phytochemical index					
*Range*	<20.9	20.9-28.3	28.4–37.1	>37.1	
*Mean*	17.4 ± 5.6	25.4 ± 6.4	32.9 ± 8.3	42.7 ± 10.7	
Energy intake *(kcal/d)*	2763 ± 26	2374 ± 24	2172 ± 24	1822 ± 25	*<0.001*
Carbohydrate *(% of total energy)*	54.7 ± 0.3	56.1 ± 0.3	58.4 ± 0.3	61.2 ± 0.3	*<0.001*
Fat *(% of total energy)*	33.3 ± *0.3*	32.6 ± 0.3	30.8 ± 0.3	28.5 ± 0.3	*<0.001*
Protein *(% of total energy)*	12.9 ± 0.1	13.4 ± 0.1	13.7 ± 0.1	14.4 ± 0.1	*<0.001*
Total fiber *(g/d)*	36.1 ± 0.8	36.1 ± 0.7	38.3 ± 0.7	41.4 ± 0.8	*<0.001*
Total carotenoids *(μg/d)*	6330 ± 303	9364 ± 267	11237 ± 263	12774 ± 294	*<0.001*
Vitamin E *(mg/d)*	10.9 ± 0.2	12.1 ± 0.2	11.7 ± 0.2	11.9 ± 0.2	*<0.001*
Vitamin C *(mg/d)*	77.3 ± 3.8	141 ± 3.4	167 ± 3.3	195 ± 3.6	*<0.001*
Whole grains *(g/d)*	7.5 ± 4.1	63.0 ± 3.6	120 ± 3.6	177 ± 4.0	*<0.001*
Fruits *(g/d)*	149 ± 11.8	365 ± 10.3	437 ± 10.2	528 ± 11.4	*<0.001*
Vegetables *(g/d)*	207 ± 9.2	284 ± 8.1	327 ± 8.0	358 ± 8.9	*<0.001*
Legumes *(g/d)*	11.8 ± 1.0	14.6 ± 0.9	17.9 ± 0.9	17.7 ± 1.0	*<0.001*
Seeds *(g/d)*	0.3 ± 0.2	1.9 ± 0.2	1.8 ± 0.2	3.1 ± 0.2	*<0.001*
Nuts *(g/d)*	3.0 ± 0.4	6.9 ± 0.4	8.7 ± 0.4	9.8 ± 0.4	*<0.001*
Olive oil *(g/d)*	0.1 ± 0.1	0.6 ± 0.1	0.9 ± 0.1	1.4 ± 0.1	*<0.001*
Soy sources *(g/d)*	1.0 ± 0.2	1.7 ± 0.1	1.6 ± 0.1	2.5 ± 0.2	*0.005*

^1^ Data are age-and energy adjusted mean ± SEM.

^2^*P* values compared the dietary intakes of participants across quartiles of dietary phytochemical index using linear regression models with adjustment of sex, age and energy intakes.

After adjustment for potential confounding variables, dietary intakes of whole grains in the highest quartile category were inversely associated with 3-year changes in weight (Table 3) and WC (Table 4) (*P for trend <0.05*). Dietary intake of fruits in the highest quartile also was associated with lower weight gain during the study period (*P for trend <0.05*) (Table 3). Inverse associations between dietary intakes of vegetables and nuts and 3-year changes in weight (Table 3), WC (Table 4) and BAI (Table 5) were stronger across quartiles categories (*P for trend <0.05*). As presented in Table 6, there was significant inverse association between highest quartile category of dietary PI and 3-year changes in weight and BAI in the study participants (*P for trend <0.05*).

**Table 3:** **The association between dietary intake of phytochemical-rich foods at baseline and 3-year change in weight**^**1**^

		**Adjusted weight change**					
	**Whole grains**	**Vegetables**	**Fruits**	**Legumes**	**Soy**	**Nuts**	**Olive and olive oils**
Q_1_ (reference)	0.0	0.0	0.0	0.0	0.0		0.0
Q_2_	−0.61 (−1.54, 0.31)	0.15 (−0.49, 0.78)	−0.77 (−1.7, 0.15)	−0.24 (−0.87, 0.39)	0.01 (−1.27, 1.29)	0.34 (−0.89, 0.96)	0.12 (−0.79, 1.02)
Q_3_	−0.16 (−1.08, 0.76)	−0.32 (−0.96, 0.32)	−0.71 (−1.63, 0.22)	0.12 (−0.51, 0.76)	0.53 (−0.28, 1.37)	−0.14 (−1.05, 0.78)	0.61 (−0.85, 0.97)
Q_4_	−1.10 (−2.02, -0.18)	−1.01 (−0.74, 0.54)	−1.19 (−2.11, -0.26)	0.13 (−0.50, 0.76)	0.59 (−0.23, 1.40)	−0.55 (−1.48, 0.37)	0.85 (−0.59, 1.76)
*P* for trend^2^	0.05	0.03	0.01	0.41	0.11	0.01	0.22

^1^Data are β regression and 95% confidence interval were estimated by using multiple regression models with adjustment for sex, age at baseline (y, continuous), BMI (kg/m^2^, continuous), education (4 categories), smoking (yes or no), physical activity (MET-h/wk, continuous), total energy intake (kcal/d), dietary carbohydrate (% of energy), fat (% of energy) and protein (% of energy).

^2^A linear trend test was performed by considering each ordinal score variable as a continuous variable in the model.

**Table 4:** **The association between dietary intake of phytochemical-rich foods at baseline and 3-year change in waist circumference**^**1**^

		**Adjusted waist circumference change**					
	**Whole grains**	**Vegetables**	**Fruits**	**Legumes**	**Soy**	**Nuts**	**Olive and olive oils**
Q_1_ (reference)	0.0	0.0	0.0	0.0	0.0	0.0	0.0
Q_2_	−0.57 (−1.15, 1.04)	1.02 (0.02, 2.03)	0.26 (−0.74, 1.28)	0.50 (−0.48, 1.49)	−0.45 (−1.85, 0.95)	0.42 (−1.49, 0.65)	−0.03 (−10.3, 0.96)
Q_3_	−0.78 (−1.88, 0.32)	0.17 (−0.87, 1.20)	0.33 (0.68, 1.35)	0.35 (−0.64, 1.34)	−0.03 (−0.92, 0.85)	−0.21 (−1.29, 0.87)	−0.34 (−1.34, 0.65)
Q_4_	−1.43 (−2.53, -0.33)	0.66 (−0.46, 1.78)	−0.27 (−1.30, 0.76)	0.54 (−0.45, 1.53)	0.47 (−0.41, 1.34)	−0.51 (−1.58, 0.56)	0.78 (−0.26, 1.81)
*P* for trend^2^	0.001	0.27	0.006	0.12	0.33	0.032	0.12

^1^Data are β regression and 95% confidence interval were estimated by using multiple regression models with adjustment for sex, age at baseline (y, continuous), BMI (kg/m^2^, continuous), education (4 categories), smoking (yes or no), physical activity (MET-h/wk, continuous), total energy intake (kcal/d), dietary carbohydrate (% of energy), fat (% of energy) and protein (% of energy).

^2^A linear trend test was performed by considering each ordinal score variable as a continuous variable in the model.

**Table 5:** **The association between dietary intake of phytochemical-rich foods at baseline and 3-year change in body adiposity index**^**1**^

		**Adjusted body adiposity index change**					
	**Whole grains**	**Vegetables**	**Fruits**	**Legumes**	**Soy**	**Nuts**	**Olive and olive oils**
Q_1_ (reference)	0.0	0.0	0.0	0.0	0.0	0.0	0.0
Q_2_	−0.69 (−1.61, 0.21)	1.03 (0.14, 1.93)	−0.48 (−1.40, 0.44)	0.24 (−0.64, 1.12)	−0.68 (−1.94, 0.58)	−0.69 (−1.61, 0.22)	0.49 (−0.39, 1.38)
Q_3_	−0.56 (−1.48, 0.35)	0.83 (−0.07, 1.73)	−0.62 (−1.55, 0.29)	0.29 (−0.59, 1.18)	−0.07 (−0.87, 0.73)	−0.28 (−1.19, 0.63)	0.10 (−0.79, 0.98)
Q_4_	−0.46 (−1.42, 0.51)	0.78 (−3.6, 1.70)	−1.36 (−2.29, 0.44)	0.53 (−0.35, 1.41)	0.52 (−0.28, 1.31)	−0.26 (−1.18, 0.67)	0.56 (−0.36, 1.50)
*P* for trend^2^	0.001	0.001	0.006	0.001	0.001	0. 45	0.22

^1^Data are β regression and 95% confidence interval were estimated by using multiple regression models with adjustment for sex, age at baseline (y, continuous), BMI (kg/m^2^, continuous), education (4 categories), smoking (yes or no), physical activity (MET-h/wk, continuous), total energy intake (kcal/d), dietary carbohydrate (% of energy), fat (% of energy) and protein (% of energy).

^2^A linear trend test was performed by considering each ordinal score variable as a continuous variable in the model.

**Table 6:** **The association between dietary phytochemical index at baseline and 3-year change in anthropometric measures**^**1**^

	**Weight change**	**Waist circumference change**	**Body adiposity change**
Q_1_ (reference)	0.0	0.0	0.0
Q_2_	−0.54 (−1.46, 0.38)	0.15 (−0.95, 1.26)	−0.79 (−1.72, 0.12)
Q_3_	−0.26 (−1.18, 0.66)	0.51 (−0.58, 1.62)	−0.40 (−1.32, 0.52)
Q_4_	−1.41 (−2.33, -0.48)	−0.05 (−1.15, 1.06)	−1.49 (−2.41, -0.56)
*P* for trend^2^	0.001	0.001	0.001

^1^Data are β regression and 95% confidence interval were estimated by using multiple regression models with adjustment for sex, age at baseline (y, continuous), BMI (kg/m^2^, continuous), education (4 categories), smoking (yes or no), physical activity (MET-h/wk, continuous), total energy intake (kcal/d), dietary carbohydrate (% of energy), fat (% of energy) and protein (% of energy).

^2^A linear trend test was performed by considering each ordinal score variable as a continuous variable in the model.

Medians of dietary phytochemical index quartiles were 17.3, 24.9, 31.8, and 41 in the first1, 2, 3 and 4^th^ quartile categories, respectively.

## Discussion

In this longitudinal study, we found that increased energy intakes, more than 37% of energy, from phytochemical rich foods could prevent weight gain and decrease body adiposity in adults during 3 year follow-up. Higher intakes of whole grains and fruits appeared to benefit more than other phytochemical-rich foods in prevention of weight and body fat gain. In our study, we observed higher intakes of phytochemical-rich foods and dietary PI in older; it suggesting that older participants are more likely to have healthy diets characterized by the consumption of plant foods. These findings in agreement with other studies, demonstrate that diet quality improved with age [28].

The mean for yearly weight gain of the participants in this study, was estimated of 0.49 kg/y over the 3 years follow-up; as compared with the higher quartile category of PI, participants in the lower quartile had twice the mean weight gain (0.64 *vs.* 0.31 kg/y), during the 3-year follow-up. Even modest weight gains have been shown to increase the risk of cardiovascular, type 2 diabetes, hypertension and other chronic diseases [29]; therefore, a beneficial effect of high dietary PI and phytochemical-rich foods intake over a 3-year period on weight gain, as observed in this study, could be instrumental in to attenuating the development of chronic disease.

The body fat, rather than the amount of excess weight, is associated with cardio-metabolic risk factors and determines the health risks of obesity, type 2 diabetes mellitus, and cardiovascular disease [30]. BAI, which estimates percentage of adiposity directly, has been recently proposed as a new and more reliable measurement enable to facilitating fat mass determination better than BMI [27,31]. In the current study, dietary PI was inversely associated with change in body adiposity during the study follow-up; participants with higher dietary PI had 1.47% decrease in BAI as compared with the reference group.

Recently, Vincent et al. [32] in a cross-sectional study of 54 healthy young adults (18–30 y) reported that PI score was inversely related to BMI, WC, waist-to-hip ratio and body fat. There are no other data in relation to dietary PI and anthropometric measures. However several previous investigations have confirmed the beneficial effects of vegetarian diets and whole plant foods to be rich sources of phytochemicals effective in body weight management [12,33,34].

In addition to dietary PI, higher intake of whole grains in this study was inversely associated with 3-year changes in weight and BAI; higher intake of fruit was also related with lower weight gain. We also found weak inverse associations between higher intakes of vegetables with lower weight gain, higher intakes of nuts with lower weight gain and WC change, and higher intakes of fruits with lower abdominal fat gain and reduction in body fat during the 3-year follow-up.

Many cross-sectional studies reported that higher intake of whole grains is related with lower BMI, and smaller WC in adults [35]; confirmed by the results of longitudinal studies one unit difference in BMI was observed between the highest and lowest whole grain intakes [36,37]. Higher intakes of whole grain foods also have been associated with lower abdominal fat as measured by dual-energy X ray absorptiometry, and subcutaneous and visceral adipose tissue volume [38,39]. In addition low glycemic index and high fiber content of whole grains, high phytochemical content including phenolic compounds (Ferulic acid, vanillic acid, caffeic acid, syringic acid, cumaric acid, anthocyanidins, quinines, flavonols, chalones, flavones and flavanones) may contribute to the beneficial effects of whole grains [40]. Fruits and vegetables are other rich sources of phytochemicals. A recent systematic review of 23 longitudinal and experimental studies indicated that higher consumption of fruits and vegetables was associated with a very gradual weight gain, and contributed to reduced adiposity in overweight and obese adults in clinical trials [41].

Our findings did not demonstrate a significant association between dietary legumes and 3-year changes in anthropometric measures, but the association between legumes and BAI positively intensified across increase quartiles of legumes intakes; a similar trend was observed for the association of soy intakes and 3-year BAI change. Despite legumes being considered as a component of a healthy diet [42], there is insufficient evidence to draw clear conclusions about the protective effect of legumes on weight [43]. Soy Isoflavones, mainly genistein, daidzein and glycitein, have been found as anti obesity factors in cell culture and animal studies; but the results of human studies are inconsistent [44,45].

Another noteworthy finding in our results was a significant trend in intensity of inverse relation between nuts intake and 3-year changes in weight and abdominal fat in adults. Nuts in addition to mono- and polyunsaturated fat are also rich sources of fiber, plant sterol, antioxidant vitamins and polyphenols may mediate multiple health benefits. Longitudinal follow-up studies, reported that frequent nut consumption was associated with a significantly reduced risk of weight gain [46,47].

There are several mechanisms that could explain the favorable effects of phytochemical-rich diets against obesity. The inverse association of phytochemicals and anthropometric measures may be mediated through their effects on decrease of appetite, regulation of carbohydrate and lipid metabolism, and direct effects on adipocytes [21]. A number of fruit and vegetable polyphenols such as quercetin, naringenin, rutin, hesperidin and resveratrol were shown to inhibit preadipocyte proliferation and induce apoptosis. Several other flavonoids also decrease adipogenesis and stimulate lipolysis in adipocytes [48]. Some phytochemicals also act as natural ligands for peroxisome proliferator-activated receptors (PPARs), and regulate lipid metabolism in the liver, promote uptake, utilization, and catabolism of fatty acids by up-regulation of genes involved in fatty acid transport and peroxisomal and mitochondrial fatty acid β-oxidation [49].

To our knowledge, this is the first longitudinal population-based study on usual dietary PI and changes in weight, WC and BAI. Use of a validated FFQ to assess usual dietary intakes, and 3 year follow-up to evaluate the anthropometric changes of the participants may be considered as strengths of this study.

Some limitations of the current study should be considered; usual dietary intakes of participants were only assessed at baseline, while several evaluations of dietary intakes could be increased the validity of the results. Using the USDA FCT rather than a complete Iranian FCT is another limitation.

## Conclusion

We found that increased dietary PI score as energy intake from phytochemical rich foods, especially whole grains and fruits, had favorable effects on prevention of weight gain and decrease of body adiposity during 3 year follow-up, as main factors contributing to development of chronic disease. Based on these results and other previous reports, definition of recommendations for food selection improvement and increase energy intakes from phytochemical rich foods is essential.

## Abbreviations

PI: Phytochemical index; WC: Waist circumference; BAI: Body adiposity index; BMI: Body mass index; TLGS: Tehran Lipid and Glucose Study; FFQ: Food frequency questionnaire; FCT: Food Composition Table; USDA: U.S. Department of Agriculture; PPARs: Peroxisome proliferator-activated receptors.

## Competing interests

The authors declare that they have no competing of interest.

## Authors’ contribution

The project was designed and implemented by Z.B and P.M. Data were analyzed and interpreted Z.B and M.G. Z.B, M.G, N.SH and F.A prepared the manuscript. P.M, and F.A supervised overall project. All authors read and approved the final version of manuscript.
